# Design of short Venturi flow meters for incompressible and isothermal flow applications

**DOI:** 10.1016/j.heliyon.2024.e29311

**Published:** 2024-04-05

**Authors:** Keith Wells, Ahmad Sharifian

**Affiliations:** School of Engineering, University of Southern Queensland, Toowoomba, QLD, 4350. Australia

**Keywords:** Venturi, Short length, Reversed flow, ISO-5167

## Abstract

The Venturi flow meter offers a range of measurement options for liquids, gas, steam, and slurries in piped systems. The main criteria for assessing Venturi performance include permanent pressure loss, discharge coefficient, relative pressure loss coefficient, and measurement accuracy. However, the extended length of Venturis, relative to other flow rate measuring instruments, can present limitations in some applications. Furthermore, the manufacturing of shorter Venturis requires less material and energy. This study addresses the challenge by developing shorter Venturi meters that adhere to established performance standards. Previous studies show that cone angles, the ratio of throat diameter to inlet diameter (β-ratio), and the throat length impact the performance of a Venturi. The scope of this research considers single-phase, incompressible and isothermal flows. The investigation focuses on the effect of cone angles for flows with Reynolds numbers ranging from 5000 to 10,000,000. Two axisymmetric Venturis, with a β-ratio of 0.7, were designed and evaluated against an ISO-5167 classical Venturi with the same β-ratio. Despite the ISO-5167 Venturi outperforming the others across the key criteria, a Venturi designed with a 40-degree convergent cone angle and a 10-degree divergent cone angle was 24.9 % shorter than the classical design and demonstrated similar performance to the ISO-5167 Venturi across Reynolds numbers from 100,000 to 10,000,000.

## 1 Introduction

Venturi meters have been employed in various industries for measuring volume flow rates since the late 19th century [1]. The Venturi effect applications extend beyond flow measurement and include a wide range of uses, including carburettor systems [2], gasifier systems [3], steam ejectors [4], spray nozzles, wind tunnels [[5], [6], [7], [8], [9]], scrubbers [10], turbines [[11], [12], [13]], the production of biodiesel [14], and the harnessing of waste kinetic energy [15]. Venturi meters offer several advantages over conventional instruments, including simplicity, reliability, and minimal pressure loss. A Venturi meter consists of convergent, throat, and divergent sections. Various Venturi meters are used in the industry, with the two primary standards being ISO-5167 and ASME MFC-3M. The performance of a Venturi meter is evaluated according to several criteria. Measurement accuracy (ΔP_1-2_) is associated with the pressure difference between the inlet and throat. Permanent pressure loss (ΔP_1-3_) is defined as the pressure difference between the inlet and outlet, and the relative pressure loss coefficient (ζ) is the ratio of this permanent pressure loss to the pressure difference between the inlet and the throat. Finally, the discharge coefficient (C_d_) represents the ratio of the actual flow to the theoretical flow rates, calculated as follows:

(1) $$Q=C_{d}\sqrt{\frac{2\left({\Delta P}_{1-2}\right)}{\rho }}\frac{A_{D}}{\sqrt{{\left(\frac{A_{D}}{A_{d}}\right)}^{2}-1}}$$

Where, Q is the volume flow rate, ρ is the fluid density, and A_D_ and A_d_ are the Venturi entry and throat cross-sectional areas, respectively.

The design of a Venturi follows standardised guidelines to minimise head loss, prevent reverse flow, and ensure high measurement accuracy. However, designs prescribed by the two standards tend to be longer and may not be suitable for applications with limited space. A short Venturi design can make it more cost-effective, lighter, and easier to install in applications where space is limited. Furthermore, manufacturing shorter Venturis would require less material and energy, provided performance criteria are not compromised. Venturi meters are available in various sizes, ranging from 1.5 mm for micro-Venturi injectors [16] to several tenths of centimetres for wind tunnels [17,18]. Oversized Venturis are manufactured for refineries, with inlet diameters of 2.7 m and lengths of 13.6 m [19]. Using flow measurement equipment with small lay lengths is often advantageous to efficiently use available space. However, designing short Venturi meters requires relatively large cone angles, which can lead to flow separation and energy loss [20].

This study aims to identify a Venturi shorter than the ISO-5167 classical meter and evaluate its performance using the previously mentioned criteria across an extensive range of Reynolds numbers. Two Venturis with a β-ratio of 0.7 were proposed, featuring a substantial convergent cone angle of 40°. The first Venturi included a divergent cone angle of 10°, aligning with recommendations from prior studies, while the second had a divergent cone angle of 20°, slightly exceeding the maximum angle typically recommended by researchers. This study exclusively examines cone angles and is limited to single-phase, isothermal, and incompressible flow applications. This research introduces notable innovations in designing and evaluating compact Venturi flow meters optimised for these conditions. The reduced size of these meters addresses a critical need in applications where spatial constraints are a significant consideration, presenting a novel solution in flow measurement technology. Based on an extensive literature review, this study stands out for its distinctive goal of shortening the length of Venturis across a broad spectrum of Reynolds numbers, ranging from 5000 to 10,000,000.

## 2 Background

Numerous studies have explored the influence of Venturi geometries on performance. Previous studies have indicated that the β-ratio, which defines the ratio of throat diameter to inlet diameter, plays a significant role in determining performance. In a study investigating the effect of variations in the converging section on Venturi scrubber performance, Jawaria Ahad et al. [21] found that in addition to the β-ratio, convergent cone angle and divergent cone angle also exert a noteworthy influence on Venturi performance. The results of their study are also confirmed by several other studies (e.g. Ref. [20]). Recent studies suggest a range of β-ratios between 0.2 and 0.8 to reduce the energy loss [22,23]. Furthermore, these studies identify acceptable θ_d_ between 7° and 15° to lower energy loss, which is sensitive to small changes in the recovery cone angle [23].

The significant effect of θ_d_ relative to θ_c_ arises from boundary layer dynamics. In the convergent section of the Venturi meter, a decrease in pressure occurs in the flow direction. As the flow accelerates, the boundary layer becomes thinner, bringing the fluid flow closer to the internal wall of the Venturi. This proximity of the flow to the surface keeps it stable and relatively free of turbulence [24]. These findings align with Liu et al. [25], who observed that negative pressure gradients help prevent flow separations. Therefore, with the convergent section less susceptible to flow separation, θ_c_ can be increased without adverse effects. The divergent region, characterised by an increase in cross-sectional area, is more prone to flow separation. While the fluid outside the boundary layer maintains enough momentum to overcome the pressure pushing against it, the flow has lower momentum and can reverse direction in response to the increasing pressure in the divergent section, which may result in flow separation [24]. Therefore, in contrast to θ_c_, θ_d_ has a more profound impact on Venturi meter performance.

The research of Fried and Idelchik [26] identifies that divergent cone angles below 7° will not experience flow separation, while White [27] suggests that angles exceeding 15° may lead to flow separation. Sparrow et al. [20] explain the inconsistency in the divergent cone angle, proposing that the Reynolds number also influences flow separation.

The recovery cone angles (θ_d_) typically used in industrial applications range from 7° to 15° [22,28]. Research by Miller [22] identified that an increase in the β-value decreases the permanent pressure loss for β-values in the range of 0.2–0.75 for a 15° recovery cone angle. However, for the Venturi meter with a 7° recovery cone angle, the permanent pressure loss decreases between the β-values of 0.2 to around 0.5 and increases as the β-value approaches 0.75. The research by Miller [22] is comparable to the studies of Omega [29], which show similar patterns of permanent pressure loss.

An experimental study conducted by Reader-Harris et al. [30] on three Venturi tubes indicates that the optimal results can be obtained with a convergent angle of 10.5°. Prior research suggests that increasing θ_d_ and θ_c_ decreases the discharge coefficient, consistent with studies on permanent pressure loss. However, the study of Prasanna et al. [31] reveals that the variation in C_d_ at a Reynolds number of 600,000 is more sensitive to changes in the θ_d_. This finding is unexpected, given that the discharge coefficient depends on pressures measured at the inlet and throat.

## 3 Methodology

This study combined computational modelling with experimental work to validate its findings. Two Venturi meters of relatively short lengths were designed and fabricated, ensuring that the cone angles and β-ratio remained within parameters suggested by prior research.

### 3.1 Experimental setup

**Fig. 1:**
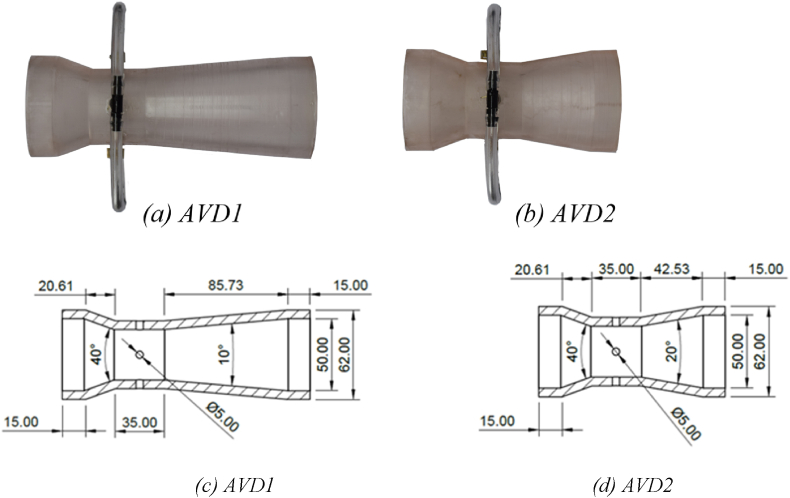
3D printed SLA resin Venturi meters a) AVD1, b) AVD2, c) AVD1 design, and d) AVD2 design.

Two axisymmetric Venturi meters, AVD1 and AVD2, with a β-ratio of 0.7, were designed and printed. Both Venturi meters featured a convergent cone angle of 40°, but their divergent cone angles were 10 and 20°, respectively. The SLA resin AVD1 Venturi meter is shown in Fig. 1a, and the SLA resin AVD2 in Fig. 1b. To comply with the ISO-5167 standard, the length and diameter of the throat of each Venturi meter were identical. Both Venturi meters featured an inlet diameter of 50 mm, with pressure tappings positioned 1D upstream and 6D downstream relative to the inlet and outlet flanges, respectively. Four pressure tappings were installed at the throat to comply with the ISO-5167 standard [28]. The internal surfaces of the SLA resin Venturi meters underwent post-processing to achieve an internal surface roughness (e) value of 2 μm, aligning with the specifications in the ISO-5167 standard. The section drawing of the AVD1 Venturi meter is illustrated in Fig. 1c, and the drawing of the AVD2 Venturi meter is in Fig. 1d. Detailed geometries of each meter are provided in Table 1.

**Table 1:** Geometries of the two Venturi flow meters used in this study.

Venturi	β	Throat Length = Throat diameter (mm)	Convergent/divergent cone angle	Total length (mm)	Change of length compared to ISO-5167 Venturi
ISO-5167	0.70	35	21°/7°	228.09	0
AVD1	0.70	35	40°/10°	171.34	−24.9 %
AVD2	0.70	35	40°/20°	128.14	−43.8 %

**Fig. 2:**
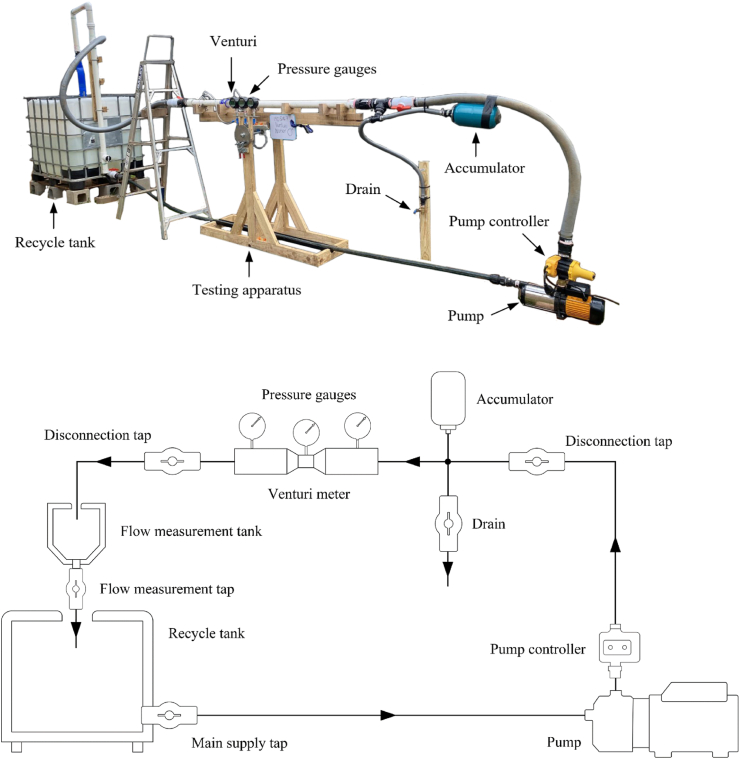
Experimental setup.

The experimental setup is shown in Fig. 2. Water was supplied by a pump capable of delivering a flow rate of 150 Lmin−1. The suction pipe of the pump was attached to the bottom of a 1000 L recycle tank. Water pressure was regulated using a pump controller, and pressure fluctuation was reduced with a pressure accumulator. The pressure gauges were positioned near the pressure tappings to reduce the amount of tubing required and to allow the pressure readings of all gauges to be captured with a digital recording device simultaneously. The outlet from the Venturi meter was connected to the top of the 1000 L recycle tank. A flow measurement system connected to the top of the 1000 L recycle tank facilitated the measurement of flow rates for each experiment.

### 3.2 Experimental uncertainties

Twenty experiments were conducted over six consecutive days, with ambient temperatures ranging from 13 °C to 24 °C. The average temperature for the twenty experiments on each Venturi meter varied marginally, falling within a range of 18 °C ± 1 °C. This slight temperature variation introduced an uncertainty of ±2.6 % in the calculations for viscosity and Reynolds number. The water level in the 1000 L recycle tank was consistently kept at approximately 500 L. Flow rate measurements were taken using a stopwatch and a 35 L measurement tank, with no significant differences observed across the experiments. However, considering the flow rate of 1.33 L/s and an estimated operator response time of ±0.5s, an anticipated uncertainty of ±2 % was factored into the flow rate measurements. Therefore, the calculated Reynolds number of 33,754 has an error of 3.3 % (2.6 %^2^+2 %^2^)^0.5^ and is presented as ≈34,000 in this article.

The digital angle meter, calibrated to an accuracy of ±0.2° by the manufacturer, was regularly verified with a water level. An additional water level was incorporated into the testing apparatus frame to ensure the testing occurred on a level surface. Given the short length of the Venturi meter, this approach resulted in a negligible uncertainty of approximately 0.1 Pa.

According to the specifications supplied by the manufacturer, the pressure gauges had an accuracy of ±0.02 % on a full scale of 30 kPa, translating to ±6 Pa uncertainty. However, the pressure gauges had a resolution of 10 Pa. Therefore, while the accuracy for individual pressure readings stood at ±10 Pa, differential pressures within the Venturi meters were accurate up to ±20 Pa.

The magnitude of pressure changes within the Venturis influences the percentage error of each experiment. For a Reynolds number of ≈34,000, measured pressure losses ranged from 160 Pa (20/160 = 12.5 %) to 240 Pa (20/240 = 8.3 %). Additional errors include uncertainties in density measurement (±0.1 %) due to temperature variations (±1 °C) and inaccuracies in the friction factor. Notably, errors in the volume flow rate effectively double since the pressure loss is proportional to the square of the volume flow rate, amplifying the error from 2 % to approximately 4 % in the pressure loss calculations. It is essential to recognise that certain components of head loss, specifically the minor head loss, are not closely related to the Reynolds number. Therefore, this calculation is anticipated to reflect the maximum possible error. Based on the Moody diagram and the Darcy-Weisbach equation for Re ≈ 34,000, the relationship between the friction factor and Reynolds number suggests a 3.3 % error could lead to around a 5 % friction factor error. Consequently, the total uncertainty ranges from 11 % (3.3 %^2^+8.3 %^2^+0.1 %^2^+5 %^2^+4 %^2^)^0.5^–14.4 % (3.3 %^2^+12.5 %^2^+0.1 %^2^+5 %^2^+4 %^2^)^0.5^.

### 3.3 Computational method

The Venturi meters were symmetrical along the centre axis, leading to axisymmetric modelling for their geometries. Creating 2D axisymmetric geometry offers the benefits of enhanced stability and faster convergence in simulations. However, it does not account for the effects of gravity and pressure tapping points.

The working fluid considered was water, with constant density and viscosity. The inlet boundary was specified as a mass flow rate boundary condition, with the flow direction normal to the boundary. The inlet turbulence intensity was 5 %, while the turbulent viscosity ratio was 10. The outlet boundary was set as a pressure outlet. The walls in the simulation were stationary with a no-slip condition, and their roughness and roughness constant were set to 2 mm and 0.5, respectively.

The discretisation mesh generated for the simulation was primarily composed of quadrilateral elements, with smaller cell sizes near the walls and ten inflation layers at a higher Reynolds number (Re > 1,000,000). The quantity of mesh elements varied for each Venturi and across various Reynolds numbers. The ISO-5167 Venturi had a maximum of 662,095 elements, the AVD1 Venturi had 845,782, and the AVD2 Venturi had 1,822,738. Table 2 displays metrics like average aspect ratio, element quality, skewness, and orthogonal quality for meshes. All these values fall within acceptable ranges and are used for flows at Re ≈ 34,000.

**Table 2:** Average characteristics of meshes used in this work at Re ≈ 34,000.

Venturi	Aspect ratio	Element quality	Skewness	Orthogonal quality	Number of mesh elements
ISO5167	1.27	0.87	0.13	0.98	209,034
AVD1	1.29	0.90	0.09	0.98	845,782
AVD2	1.29	0.85	0.15	0.97	48,091

**Fig. 3:**
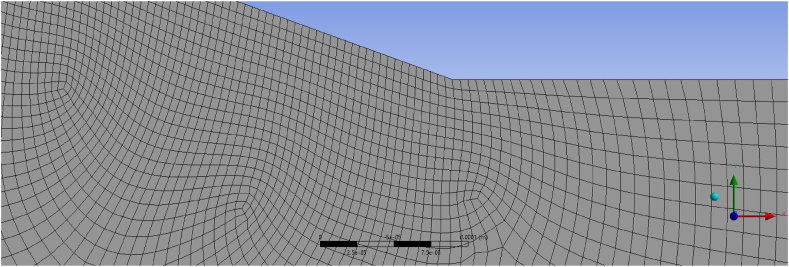
AVD1 mesh configuration at Re ~ 34000 focussing on the intersection of the convergent section and the throat of the Venturi.

Fig. 3 displays the mesh configuration for the AVD1 Venturi with 845,782 elements at the intersection of the convergent and throat sections. The mesh size in this corner is approximately 0.02 mm at the walls.

The turbulent flow approach utilised the k-omega-SST model, and a pressure-based solver was employed to investigate the fluid flow behaviour. The velocity formulation adopted was absolute, and the simulation was conducted in an axisymmetric and steady-state manner. Second-order upward schemes were utilised for discretising the pressure, momentum, turbulent kinetic energy, and specific dissipation rate. The convergence criterion for residuals of continuity, velocity components, turbulent kinetic energy (k) and specific dissipation rate (omega) was set at 1 x 10^−8^.

### 3.4 Verification of computational results

This study considered the parameters Y^+^ and GCI (Grid Convergence Index) to ensure accurate computational results. For low Reynolds numbers, a target Y^+^≤ 5 was adopted, as recommended by Sharp et al. [23] and Versteeg and Malalasekera [32]. Conversely, for higher Reynolds numbers, specifically at 10,000,000, a Y^+^≤ 300 was deemed acceptable, aligning with the recommended values for the k-omega-SST turbulence model [33]. Furthermore, to ensure grid independence of the simulations, a maximum GCI of 5 % was applied, a criterion supported by various references, including [32].

The parameters for calculating the Grid Convergence Index calculation and Y^+^ at a Reynolds number of ≈34,000 for the three Venturis are outlined in Table 3. Three mesh sets (coarse, medium and fine) were employed for the GCI calculation, incorporating a safety factor of 1.25. Table 3 confirms that all set criteria were met.

**Table 3:** GCI calculation details for AVD1, AVD2, and ISO-5167 Venturis at a Reynolds number of ≈34,000.

Venturi	Number of elements	(P_1_–P_2_) (kPa)	(P_1_–P_3_) (kPa)	C_d_	ζ	Y^+^	GCI (P_1_–P_2_)	GCI (P_1_–P_3_)	GCI C_d_	GCI ζ
ISO-5167	44352	779.0	156.3	0.965	0.201	5.17	0.03 %	4.1 %	0.01 %	0.03 %
	53175	779.0	154.9	0.965	0.200	5.44				
	209034	779.1	157.0	0.964	0.202	3.63				
AVD1	52552	776.9	158.7	0.966	0.204	4.85	0.2 %	3.6 %	0.1 %	3.4 %
	413521	776.2	156.3	0.967	0.201	1.62				
	845782	777.4	160.0	0.966	0.206	2.49				
AVD2	85675	778.5	241.7	0.965	0.310	5.0	0.1 %	0.96 %	1 %	0.05 %
	221359	776.1	227.0	0.966	0.292	2.25				
	263737	776.0	227.1	0.966	0.293	2.09				

For Reynolds numbers exceeding 1,000,000, maintaining a Y+ value around five was not possible, leading to an acceptable compromise at a value of 300. The results for a Reynolds number of 10,000,000 are presented in Table 4.

**Table 4:** GCI calculation details for AVD1, AVD2, and ISO-5167 Venturis at a Reynolds number of ≈10,000,000.

Venturi	Number of elements	(P_1_–P_2_) (MPa)	(P_1_–P_3_) (MPa)	C_d_	ζ	Y^+^	GCI (P_1_–P_2_)	GCI (P_1_–P_3_)	GCI C_d_	GCI ζ
ISO-5167	209,034	65.2	6.1	0.988	0.094	622	0.2 %	4.2 %	0.02 %	4 %
	318,348	65.0	5.7	0.990	0.088	46				
	662,095	65.0	5.8	0.990	0.090	37.1				
AVD1	52,552	64.8	5.7	0.991	0.088	1054	0.11 %	3.2 %	0.06 %	3.1 %
	413,521	64.7	5.4	0.991	0.085	159.1				
	845,782	64.7	5.6	0.991	0.087	242.2				
AVD2	263,737	64.5	7.0	0.993	0.109	290.9	0.00 %	0.3 %	0.07 %	0.2 %
	422,889	64.5	7.0	0.993	0.109	187				
	1,822,738	64.5	7.0	0.993	0.109	247.1				

### 3.5 Validation of computational results

Ensuring the accuracy and reliability of the computational results required an extensive validation process. Computational outcomes for the AVD1 and AVD2 Venturis were compared with experimental data obtained at a Reynolds number of ≈34,000. For the ISO-5167 Venturi, this validation involved comparing the values provided by the standard across a broad spectrum of Reynolds numbers.

The ISO-5167 standard provides discharge coefficients and ζ-ratios for various scenarios, as listed in Table 5. These were compared with the data specified in the standard. While the standard provides discharge coefficients for different manufacturing processes, it omits specific values for printed Venturis. Nonetheless, Table 5 demonstrates that the impact of the manufacturing process is relatively minimal. The computational results for flows at varying Reynolds numbers closely align with the benchmarks set by ISO-5167, confirming the reliability of the computational results.

**Table 5:** Comparison of computational outcomes for the ISO-5167 Venturi with a beta ratio of 0.7 to results provided by ISO-5167 Standard - Part 4, Appendices B and C, across varied Reynolds numbers.

Manufacturing process	Variable	Reynolds range	Value	Uncertainty	Computational results
Classical Venturi tube with an “as cast” convergent section	C_d_	100,000	0.976	1.5 %	0.97
Classical Venturi tube with a machined convergent section	C_d_	50,000	0.970	3 %	0.97
		100,000	0.977	2.5 %	0.97
		200,000	0.992	2.5 %	0.98
		500,000–1,000,000	0.995	1 %	0.98-0.99
		1,000,000–2.000,000	1.000	2 %	0.99-0.99
		2,000,000–100,000,000	1.010	3 %	0.99-0.99
Classical Venturi tube with a rough-welded sheet-iron convergent section	C_d_	100,000	0.98	2.5 %	0.97
Classical Venturi tube with a profile as defined for an “as cast” convergent section but with the entrance cylinder and convergent section machined	C_d_	10,000	0.963	2.5 %	0.94
		100,000	0.980	1.5 %	0.97
		200,000–500,000	0.992	1 %	0.98-0.98
		500,000–3,200,000	0.995	1 %	0.98-0.99
a classical Venturi tube	ζ	>1,000,000	0.06-0.10	NA	0.10

The computational ζ value lies within the upper boundary defined by the standard. The standard sets the upper limit of ζ for the Venturis with the highest relative roughness associated with the Venturis with the smallest inlet diameter. In this study, all Venturis featured an inlet diameter of 50 mm, aligning with the minimum diameter prescribed by the standard. Thus, it can be concluded that the computational results for ISO-5167 Venturi are in good agreement with the values provided by the standard.

Table 6 presents comparative data for Venturis AVD1 and AVD2 at Re ≈ 34,000. The computational and experimental results for the permanent pressure loss (ΔP_1-3_) of AVD1 closely align, while the ΔP_1-2_ is slightly higher than the computational value. Notably, the computational values for ΔP_1-2_ and ΔP_1-3_ for AVD2 are 20 % and 23 % less than the experimental results. These variances arise from not modelling pressure tapping and due to notable flow fluctuations, as well as flow separation and reversed flow, observed in the AVD2 Venturi during experiments. It is pertinent to note that the displayed results represent average values from twenty experiments. The results of the twenty experiments demonstrate significant variance, with ΔP_1-2_ ranging from 0.59 kPa to 1.35 kPa and ΔP_1-3_ fluctuating between −0.12 kPa and 0.8 kPa.

**Table 6:** Comparison of experimental and computational results for AVD1 and AVD2 Venturis at Re = 34,000.

Venturi	(P_1_–P_2_) (CFD)(kPa)	(P_1_–P_3_) (CFD) (kPa)	No. mesh elements	(P_1_–P_2_) (EXP)(kPa)	(P_1_–P_3_) (EXP)(kPa)
AVD1	0.78	0.16	845782	0.81	0.16
AVD2	0.78	0.23	48091	0.97	0.30

After excluding the results of ten experiments deemed to be outliers, the average experimental values are refined to 0.93 kPa for ΔP_1-2_ and 0.27 kPa for ΔP_1-3_, aligning them more closely with the computational findings. Nonetheless, compared to the experimental outcomes, the computational results remain 15 % less for ΔP_1-2_ and 16 % less for ΔP_1-3_. The experimental data and the observed fluctuations suggest that the AVD2 Venturi might not be optimal for flow measurements, especially at Re ≈ 34,000.

The computational results align well with the ISO-5167 standard across all Reynolds numbers. Similarly, a strong correlation exists between the experimental and computational data for AVD1. However, for AVD2, a significant discrepancy of 16 % arises compared to the experimental data, surpassing the maximum estimated experimental error of 11 % for this Venturi. The results section will explain factors contributing to the relatively high uncertainties in the experimental results for AVD2. Considering the factors discussed, the computational results can be deemed validated and reliable.

## 4 Results and discussion

This section presents the computational results of all three Venturis for Reynolds numbers from 5000 to 10,000,000. These results are classified into flow patterns, ΔP_1-2_, ΔP_1-3_, ζ and C_d_.

### 4.1 Flow pattern

The flow pattern within a Venturi meter is crucial because it influences measurement accuracy and reliability and offers valuable visual insights into flow behaviours. It should be noted that this study could not capture cavitation or choking as constant density, single-phase and isothermal processes were assumed.

**Fig. 4:**
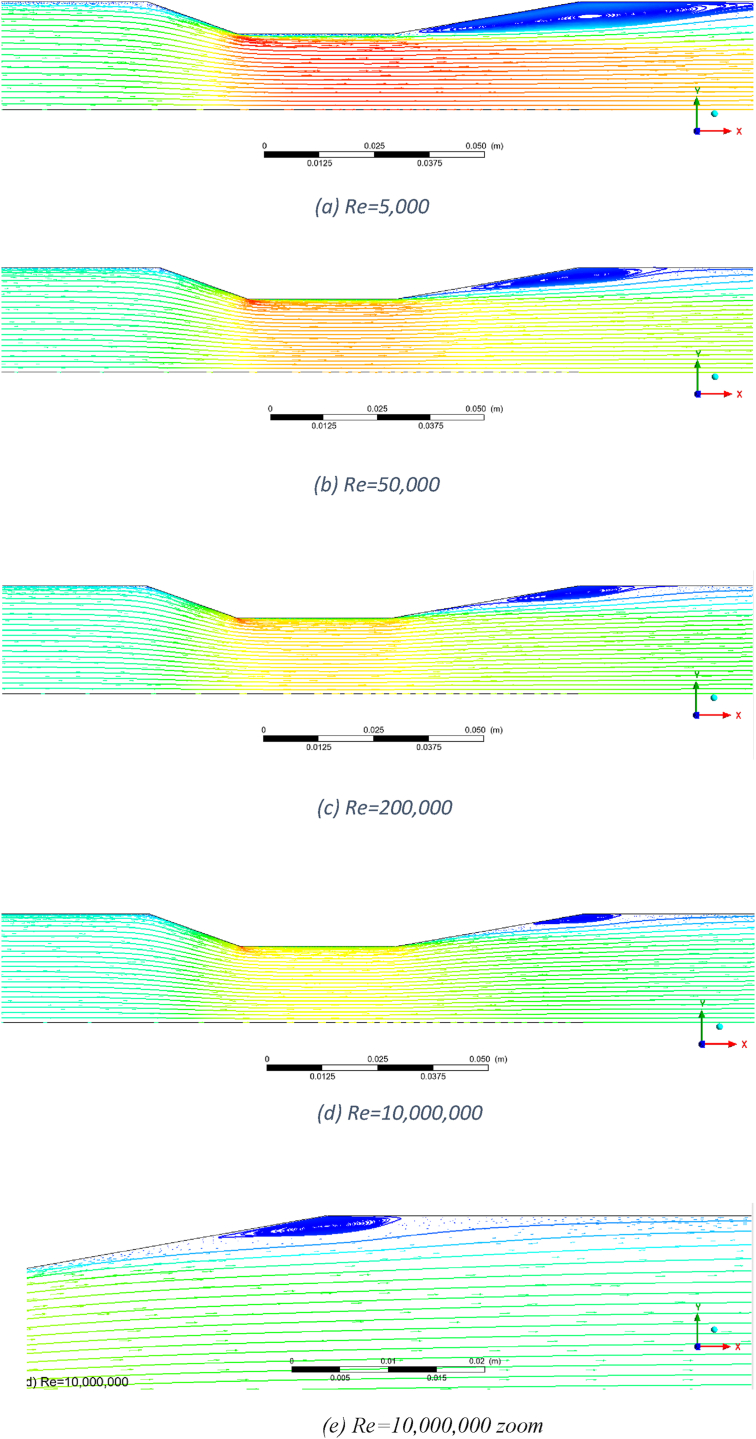
Flow patterns for AVD2 Venturi in a) Re = 5000, b) Re = 50,000, c) Re = 200,000, d) Re = 10,000,000 and e) Re = 10,000,000 zoom.

The flow pattern for the AVD2 Venturi at different Reynolds numbers is presented in Fig. 4. This Venturi exhibits flow separation and reverse flow across all Reynolds numbers studied in this work. Fig. 4a demonstrates a prominent reverse flow that initiates from the start of the divergent section and extends beyond the divergent section at a Reynolds number of 5000. There are two reasons for this pronounced reverse flow. The first reason is the high divergent cone angle (20°), and the second is the relatively low velocity. Due to insufficient momentum, the flow cannot overcome the adverse pressure gradient in the divergent section, leading to detachment from the wall. As the Reynolds number increases (see Fig. 4b,c & d), the flow does not follow the wall due to the high divergent cone angle, but as the flow momentum increases, the separation from the wall starts later, closer to the midpoint of the divergent section. The size of the reversed flow region diminishes with the increase of the Reynolds number, yet it exists even at Re = 10,000,000. Fig. 4e provides a zoomed-in view of this flow behaviour at Re = 10,000,000, highlighting the detailed flow structures and the extent of flow separation.

In conjunction with generated eddies, this reversed flow is anticipated to increase permanent pressure loss and introduce pressure fluctuations due to the inherently stochastic nature of turbulence and eddies. For Reynolds numbers exceeding 200,000, the eddies are absent at the throat or the exit pressure tapping point (6D from the end of the Venturi). In practical flow measurement applications, the exit pressure tapping point often does not exist, and only pressure at point 1 (the inlet pressure tapping point) and point 2 (the pressure tapping point at the midpoint of the throat) are measured. Nonetheless, residual fluctuations from eddies at the divergent section may still show at those points, as observed in the experimental work of this study (see section 3.5). Consequently, this Venturi is not recommended for the Reynolds number range of 5000 to 10,000,000.

**Fig. 5:**
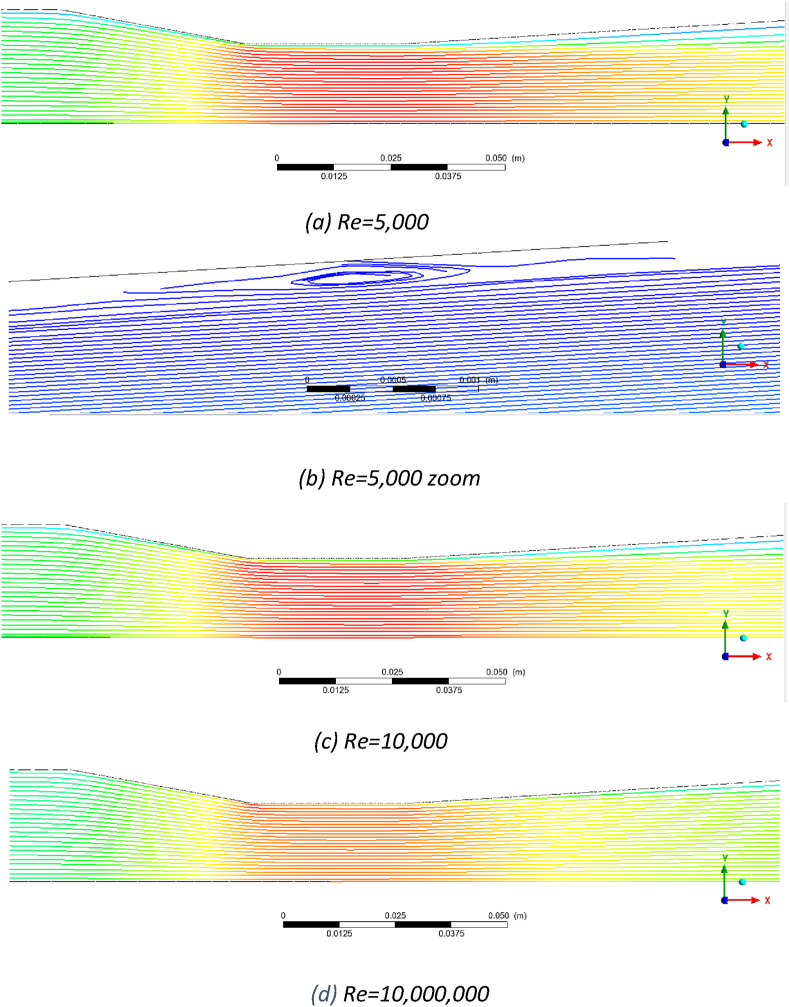
Flow pattern in Venturi ISO-5167 a) Re = 5000, b) Re = 5,000, zoom, c) Re = 10,000, and d) Re = 10,000,000

The ISO-5167 Venturi exhibits the most favourable flow pattern, as depicted in Fig. 5. Fig. 5a demonstrates that the Venturi flow is streamlined for a Reynolds number of 5000. However, upon closer investigation, small eddies and reversed flow were detected within the middle of the divergent section (Fig. 5b). The diameters of these eddies are small and on the order of fractions of a millimetre. This reversed flow shows a low velocity of 1 mm/s and does not appear to influence the main flow within the Venturi. In real-world applications, these small turbulence phenomena are insubstantial, as they are not expected to cause noticeable pressure fluctuations at the pressure tapping points within the middle of the throat section (point 2) or the tapping point located at the entrance point of the Venturi (point 1).

In Fig. 5c, with an increase in Reynolds number to 10,000, the flow pattern maintains its streamlined nature, and further investigation reveals the absence of small eddies. This observation shows a suitable selection of the cone divergent angle. Small eddies at a Reynolds number of 5000 in the boundary layer are likely a result of inadequate flow momentum to overcome the increasing pressure gradient in the divergent section. Meanwhile, Fig. 5c still indicates the presence of low-velocity regions adjacent to the walls, even though the flow does not reverse. As Reynolds numbers rise, the flow has adequate momentum, reducing the low-velocity regions near the Venturi walls. Lastly, Fig. 5d presents the flow pattern at a Reynolds number of 10,000,000, showing the absence of reversed flow and the eddies within the Venturi.

**Fig. 6:**
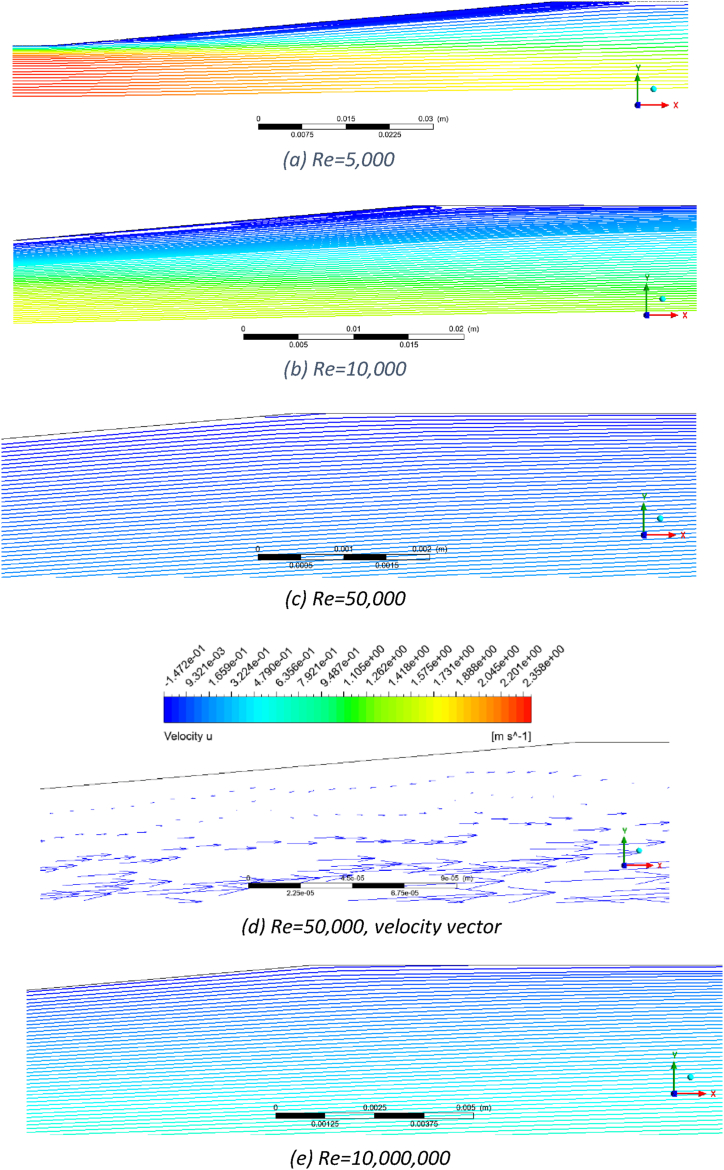
AVD1 Venturi, a) Re = 5000, b) Re = 10,000, c) Re = 50,000, d) Re = 50,000, velocity vector, and e)Re = 10,000,000.

Fig. 6 demonstrates the flow pattern for the AVD1 Venturi across varying Reynolds numbers. In Fig. 6a, at a Reynolds number of 5,000, a region of reversed flow with substantial size is evident along the wall of the divergent section. While this area of reversed flow is smaller than AVD2, it is notably larger than the one observed in the ISO-5167 Venturi. This outcome aligns with expectations since all three Venturis possess the same β-ratio, but the divergent cone angle (10^⸰^) of AVD1 falls between ISO-5167 (7^⸰^) and AVD2 (20^⸰^). Due to this pronounced reversed flow region, the AVD1 Venturi should not be used for flow with a Reynolds number of 5000. As the Reynolds number increases to 10,000, the extent of the reverse flow region diminishes, as depicted in Fig. 6b. The reversed flow region decreases at Re = 50,000 (Fig. 6c) as the momentum overcomes the positive pressure gradient. At Reynolds numbers of 100,000 and beyond, the flow becomes streamlined without any reversed flow, as shown in Fig. 6d and further confirmed at a Reynolds number of 10,000,000 in Fig. 6e.

### 4.2 Performance criteria

**Fig. 7:**
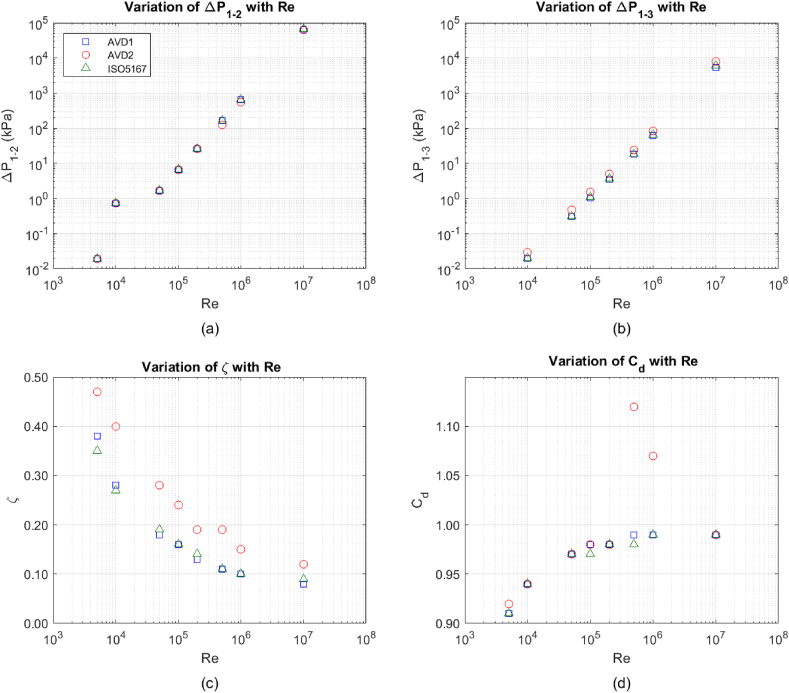
The computational results for Venturis AVD1, AVD2 and ISO-5167 a) ΔP_1-2_ versus Re, b) ΔP_1-3_ versus Re, c) ζ versus Re, and d) C_d_ versus Re.

In this section, the calculated values of (ΔP_1-2_) and (ΔP_1-3_), their ratios (ζ) and discharge coefficients (C_d_) for all three Venturis are presented at Reynolds numbers ranging from 5000 to 10,000,000. The results are presented in Fig. 7 and Table 7.

**Table 7:** The computational results for the AVD1, AVD2 and ISO-5167 Venturis at varying Reynolds numbers.

	ΔP_1-2_ (kPa)			ΔP_1-3_ (kPa)			C_d_			ζ		
Re	AVD1	AVD2	ISO5167	AVD1	AVD2	ISO5167	AVD1	AVD2	ISO5167	AVD1	AVD2	ISO5167
5000	0.019	0.019	0.019	0.007	0.009	0.007	0.91	0.92	0.91	0.38	0.47	0.35
10000	0.72	0.72	0.73	0.020	0.029	0.020	0.94	0.94	0.94	0.28	0.40	0.27
50000	1.7	1.7	1.7	0.308	0.477	0.315	0.97	0.97	0.97	0.18	0.28	0.19
100000	6.6	6.6	6.7	1.03	1.57	1.09	0.98	0.98	0.97	0.16	0.24	0.16
200000	26.3	26.3	26.5	3.50	5.03	3.69	0.98	0.98	0.98	0.13	0.19	0.14
500000	162.8	126.7	163.8	18.04	24.27	18.33	0.99	1.12	0.98	0.11	0.19	0.11
1000000	648.1	551.5	652.2	62.1	84.0	65.1	0.99	1.07	0.99	0.10	0.15	0.10
10000000	64.7 × 10^3^	64.7 × 10^3^	65.2 × 10^3^	5.44 × 10^3^	7.95 × 10^3^	6.11 × 10^3^	0.99	0.99	0.99	0.08	0.12	0.09

Fig. 7a illustrates that the calculated values of (ΔP_1-2_) for the three Venturis increase with the Reynolds number, as anticipated. While the values for these Venturis are generally similar, discrepancies emerge at Reynolds numbers of 500,000 and 1,000,000. Notably, the AVD2 Venturi significantly drops in measurement accuracy (ΔP_1-2_) at these Reynolds numbers. Among all the Reynolds numbers, the ISO-5167 Venturi has a marginally superior pressure accuracy compared to the AVD1 Venturi. Given that the AVD1 and AVD2 Venturis share the same convergent cone angle and β-ratio, the primary distinguishing factor causing these observed variances is likely the impact of reversed flow. Such flow increases throat pressure, leading to a decrease in ΔP_1-2_.

Fig. 7b presents the calculated values of the permanent pressure loss (ΔP_1-3_). As expected, the permanent pressure loss of the AVD2 Venturi for all Reynolds numbers is more significant than the two other Venturis due to strong reversed flow. The permanent pressure loss of the AVD1 Venturi is similar to the ISO-5167 for Reynolds numbers lower than 50,000 but less than the ISO-5167 for higher Reynolds numbers, and the difference increases with the Reynolds number. A similar permanent pressure loss for low Reynolds numbers may be due to the reversed flow observed in both Venturi meters. This phenomenon is further influenced by the slightly greater divergent cone angle of AVD1, which is expected to increase the pressure loss. Additionally, the shorter length of AVD1 (171.34 mm), in contrast to the ISO-5167 Venturi (228.09 mm), is expected to decrease the pressure loss. The overall outcome of the above variables for low Reynolds numbers is similar to the permanent pressure losses of the two Venturis.

As the Reynolds number increases to 50,000 and above, the size of the reversed flow region for the AVD1 Venturi decreases and disappears, as shown in Fig. 6. Thus, for a Reynolds number ≥100,000, the permanent pressure loss exhibited by the AVD1 Venturi is marginally lower compared to the ISO-5167. This observation can be attributed to the absence of reversed flow in the AVD1 Venturi and reduced friction loss resulting from its shorter length.

The critical parameter for selecting a Venturi for most applications is ζ, detailed in Table 7 and illustrated in Fig. 7c. The AVD2 demonstrates considerably greater ζ compared with the two other Venturis. The ζ-ratio values of AVD1 and ISO-5167 are similar. The ISO-5167 outperforms the AVD1 for Reynolds numbers ≤50,000, but the AVD1 Venturi performs slightly better for higher Reynolds numbers. This decreasing trend of the ζ-ratio with an increase in Reynolds number aligns with expectations and is consistent with findings from a previous study [28].

The discharge coefficients of the Venturis, illustrated in Fig. 7d and tabulated in Table 7, demonstrate an increase corresponding to a rise in Reynolds number, a correlation well-documented in prior studies [28]. The discharge coefficient of AVD2 is consistent with this trend and remains comparable to those of the other two Venturis, except for Reynolds numbers 500,000 and 1,000,000, where it exceeds the value of one. This trend could be expected as (ΔP_1-2_) for this Venturi is influenced by the reversed flow. Equation (1) shows that the decrease of (ΔP_1-2_) for a known Q causes the C_d_ to increase. For a high Reynolds number of 10,000,000, depicted in Fig. 7d, the reversed flow region relocates from point 2 (middle of the throat), as illustrated in Fig. 4d, diminishing its impact on the discharge coefficient. It is worth noting that in practical applications, the permanent pressure loss holds greater significance than the value of C_d_, as C_d_ is solely influenced by (ΔP_1-2_). Maintaining a relatively constant C_d_ across varying Reynolds numbers is advantageous for a Venturi. However, AVD1 and ISO-5167 Venturis both exhibit comparable measurement accuracy, with their discharge coefficient shifting from 0.91 at Re = 5000 to 0.99 at Re = 10,000,000. The ISO-5167 and AVD1 Venturis demonstrate comparable performance in terms of their discharge coefficients.

## 5 Further discussion

Previous research established that the convergent cone angle exerts minimal influence on Venturi performance, leading to the design of two Venturis with a large convergent cone angle of 40°. One of these Venturis (AVD1) features a divergent cone angle of 10°, aligning with the recommended range of 7–15° from existing studies, while the other Venturi (AVD2) possesses a larger divergent cone angle of 20°. Consequently, the AVD1 and AVD2 Venturis are 25 % and 44 % shorter, respectively, than the ISO-5167 Venturi.

The target GCI (≤0.05) and residuals (10^−8^) for verifying the computational results were attained across all Reynolds numbers for the three Venturis. Y^+^ values below five were achieved only at lower Reynolds numbers. Notably, at higher Reynolds numbers, the AVD2 Venturi, with Y^+^ values exceeding five, demonstrated reverse flow, suggesting it is an unsuitable Venturi meter. However, no reverse flow was observed for the other two Venturis for Reynolds numbers exceeding 100,000, and the results were verified, even with Y^+^ values above five. This verification is justifiable for two main reasons. Firstly, reverse flow can be attributed to the low momentum of flows in the boundary layer adjacent to the divergent section walls, particularly when the flow is subjected to an increasing pressure gradient. With increasing Reynolds numbers, the flow momentum also increases, eliminating the possibility of reverse flow. Secondly, the mesh size in the divergent section of these two Venturis for high Reynolds numbers was in the order of micrometres. Thus, even if vortices were to be generated in the flow, their diameters, being in the order of micrometres, would not significantly disrupt the main flow. Considering all these factors, the results can be confidently deemed verified.

The flow pattern performance demonstrated by the ISO-5167 Venturi was superior across all Reynolds numbers. Even for a notably low Reynolds number of 5,000, the main flow remained streamlined, with small eddies observed at the end of the divergent section, which is unlikely to influence the Venturi performance. It is concluded that the ISO-5167 Venturi is suitable for flows with Reynolds numbers ranging from 10,000 to 10,000,000 and can be cautiously employed for Reynolds numbers between 5,000 and 10,000.

AVD2, the shortest Venturi examined, exhibited suboptimal performance across several criteria. Consequently, it is not recommended for flows with Reynolds numbers ranging from 5000 to 10,000,000. Notably, the performance of AVD2 improved for very high Reynolds numbers, suggesting potential applications for Re > 10,000,000, an aspect not explored in this study.

AVD1, which is 25 % shorter than the ISO-5167 Venturi, displayed comparable performance in measurement accuracy, permanent pressure loss, relative permanent pressure loss, and discharge coefficient. Nonetheless, it exhibited reversed flow patterns for flows with Re ≤ 50,000, making it unsuitable in the low Reynolds range due to potential measurement inconsistencies. AVD1 demonstrated marginally superior performance regarding permanent pressure loss and relative permanent pressure loss for higher Reynolds numbers compared to the ISO-5167 Venturi. The superior performance of AVD1 in terms of permanent pressure loss at higher Reynolds numbers can be attributed to the greater length and larger surface area of the ISO-5167 Venturi, making it more prone to the effects of surface roughness.

In practical applications where space is limited, using a longer Venturi meter often necessitates shorter straight sections in the inlet and outlet pipes, potentially leading to the need for bends in the piping. Such bends can impact the efficiency and pressure loss characteristics of the Venturi meter, a crucial factor to consider when comparing the permanent pressure loss of different Venturis. The pressure loss in a pipe with an inlet diameter of 50 mm, a surface roughness of 2 μm, and a length differential of 56.75 mm (resulting from 228.09 mm to 171.34 mm Venturi lengths) was calculated using the Darcy-Weisbach and Colebrook equations across various Reynolds numbers. This calculated loss was then added to the permanent pressure loss of the AVD1 Venturi. The findings indicate that the pressure loss of the AVD1 Venturi matches the ISO-5167 Venturi at Re = 100,000 (1.09 kPa). However, at Re = 1,000,000, the permanent loss of AVD1 (66.0 kPa) slightly exceeded that of the ISO-5167 (65.1 kPa).

In conclusion, while factors such as surface roughness and location affect the performance of AVD1 relative to the ISO-5167 Venturi, the differences are generally minor. Notably, their performance aligns closely when Reynolds numbers are above 100,000.

## 6 Conclusion

The study evaluated the performance of three Venturis for single-phase, incompressible, and isothermal flows across a Reynolds number range of 5000 to 10,000,000. The primary objective was to identify the most compact Venturi that optimally balances measurement accuracy, permanent pressure loss, relative pressure loss, and discharge coefficient. The well-established ISO-5167 Venturi with a β-ratio of 0.7 was used as the reference for comparison.

The findings from the study include.

1. The ISO-5167 Venturi, with a β-ratio of 0.7, demonstrated excellent performance across a Reynolds number range from 10,000 to 10,000,000. This extends the recommended range specified in the ISO-5167 standard from 200,000 to 2,000,000. The study also suggests that this Venturi can be employed cautiously for lower Reynolds numbers from 5000 to 10,000.
2. The study identified a Venturi featuring a β-ratio of 0.7, a convergent cone angle of 40°, and a divergent cone angle of 10°. Despite being 24.9 % shorter than the ISO-5167 Venturi, it exhibited strong performance across a Reynolds number range of 100,000 to 10,000,000, satisfying all performance criteria.

## Funding sources

This research did not receive any specific grant from funding agencies in the public, commercial, or not-for-profit sectors.

## Data availability

Data will be made available on request.

## CRediT authorship contribution statement

**Keith Wells:** Writing – review & editing, Writing – original draft, Validation, Software, Methodology, Investigation, Formal analysis, Data curation, Conceptualization. **Ahmad Sharifian:** Writing – review & editing, Writing – original draft, Validation, Supervision, Software, Methodology, Formal analysis, Data curation, Conceptualization.

## Declaration of competing interest

The authors declare that they have no known competing financial interests or personal relationships that could have appeared to influence the work reported in this paper.

## References

[1] Cascetta F. (1995). Short history of the flowmetering. ISA Trans..

[2] Moh’d A.Q., Asfar K.R., Ramzi A.A. (2001).

[3] Pandey B., Sheth P.N., Prajapati Y.K. (2022). Air-CO2 and oxygen-enriched air-CO2 biomass gasification in an autothermal downdraft gasifier: experimental studies. Energy Convers. Manag..

[4] Tang Y., Liu Z., Li Y., Shi C., Wu H. (2017). Performance improvement of steam ejectors under designed parameters with auxiliary entrainment and structure optimisation for high energy efficiency. Energy Convers. Manag..

[5] Gato L.M.C., Carrelhas A.A.D., Cunha A.F.A. (2021). Performance improvement of the axial self-rectifying impulse air-turbine for wave energy conversion by multi-row guide vanes: design and experimental results. Energy Convers. Manag..

[6] Sharifian A., Hashempour J. (2016). A novel ember shower simulator for assessing performance of low porosity screens at high wind speeds against firebrand attacks. J. Fire Sci..

[7] Sharifian A., Hashempour J. (2020). Wind tunnel experiments on effects of woven wire screens and buffer zones in mitigating risks associated with firebrand showers. Aust. J. Mech. Eng..

[8] Hashempour J., Sharifian A. (2017). Effective factors on the performance of woven wire screens against leaf firebrand attacks. J. Fire Sci..

[9] Sharifian Barforoush A., Du Preez M. (2022). Quantifying the effectiveness of a mesh in mitigating burning capabilities of firebrand shower. Fire.

[10] Ali M., Yan C., Sun Z., Gu H., Mehboob K. (2013). Dust particle removal efficiency of a venturi scrubber. Ann. Nucl. Energy.

[11] Shaaban S., Hafiz A.A. (2012). Effect of duct geometry on Wells turbine performance. Energy Convers. Manag..

[12] Hassanpour M., Azadani L.N. (2021). Aerodynamic optimisation of the configuration of a pair of vertical axis wind turbines. Energy Convers. Manag..

[13] Abdelghafar I., Refaie A.G., Kerikous E., Thévenin D., Hoerner S. (2023). Optimum geometry of seashell-shaped wind turbine rotor: maximising output power and minimising thrust. Energy Convers. Manag..

[14] Goh B.H.H., Chong C.T., Ge Y., Ong H.C., Ng J.H., Tian B., Ashokkumar V., Lim S., Seljak T., Józsa V. (2020). Progress in utilisation of waste cooking oil for sustainable biodiesel and biojet fuel production. Energy Convers. Manag..

[15] Hunt J.D., Byers E., Prenner R., de Freitas M.A.V. (2018). Dams with head increaser effect: harnessing potential and kinetic power from rivers with large head and flow variation. Energy Convers. Manag..

[16] Degenhardt S., Cheriguen Y., Geiling T., Hoffmann M. (2016). Micro-Venturi injector: design, experimental and simulative examination. J. Phys. Conf..

[17] Sharifian A., Hashempour J. (2016). A novel ember shower simulator for assessing performance of low porosity screens at high wind speeds against firebrand attacks. J. Fire Sci..

[18] Sharifian Barforoush A., Du Preez M. (2022). Quantifying the effectiveness of a mesh in mitigating burning capabilities of firebrand shower. Fire.

[19] Matt Ligon (2020). https://blog.wika.us/products/flow-products/wika-delivers-oversized-venturi-tube-in-the-midst-of-a-pandemic/.

[20] Sparrow E.M., Abraham J.P., Minkowycz W.J. (2009). Flow separation in a diverging conical duct: effect of Reynolds number and divergence angle. Int. J. Heat Mass Tran..

[21] Ahad J., Farooq A., Siddique W., Ahmed A., Ahmad M., Waheed K., Qureshi K.R., Irfan N. (2022). Influence of variation in converging section and orifice plane on the performance of venturi scrubber by using CFD. Prog. Nucl. Energy.

[22] Miller R.W. (1996).

[23] Sharp Z.B., Johnson M.C., Barfuss S.L. (2018). Optimising the ASME Venturi recovery cone angle to minimise head loss. J. Hydraul. Eng..

[24] Wu H., Xu Y., Xiong X., Mamat E., Wang J., Zhang T. (2020). Prediction of pressure drop in Venturi based on drift-flux model and boundary layer theory. Flow Meas. Instrum..

[25] Liu P., Liu H., Yang Y., Wang M., Sun Y. (2020). Comparison of design methods for negative pressure gradient rotary bodies: a CFD study. PLoS One.

[26] Fried E., Idelchik I.E. (1989).

[27] White F.M. (2008).

[28] International Organization for Standardization (2003). Measurement of fluid flow by means of pressure differential devices inserted in circular cross-section conduits running full — part 4: venturi tubes. https://www.iso.org/standard/30192.html.

[29] Omega (2001).

[30] Reader-Harris M.J., Brunton W.C., Gibson J.J., Hodges D., Nicholson I.G. (2001). Discharge coefficients of Venturi tubes with standard and non-standard convergent angles. Flow Meas. Instrum..

[31] Prasanna M.A., Seshadri V., Kumar Y. (2016). Numerical analysis of compressible effect in the flow metering by classical venturi meter. International Journal of Engineering Sciences & Research Technology.

[32] Versteeg H.K., Malalasekera W. (2007).

[33] Lacombe F., Pelletier D., Garon A. (2019). AIAA Scitech 2019 Forum.

